# Supplementary material for: Signature of magnetic-dependent gapless odd frequency states at superconductor/ferromagnet interfaces
Source: Nat Commun. 2015 Sep 2;6:8053. doi: 10.1038/ncomms9053 (PMC4569701; doi:10.1038/ncomms9053)
Supplement: Supplementary Information — Supplementary Figures 1-9, Supplementary Note 1-2 and Supplementary References [file ncomms9053-s1.pdf]

Supplementary Figures

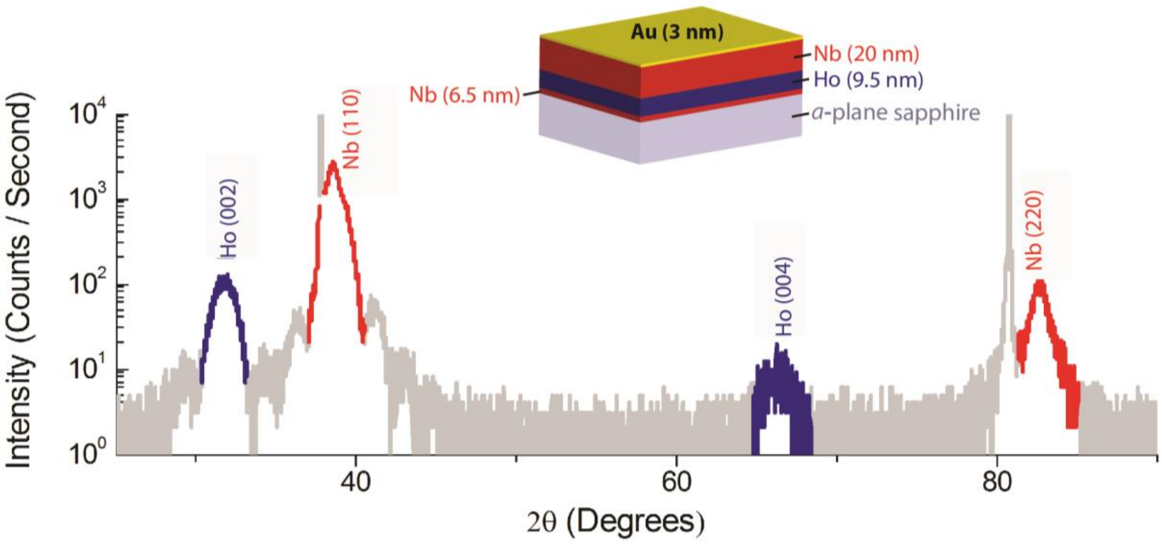

Supplementary Figure 1. High angle X-ray diffraction data of Au(3 nm)/Nb(20 nm)/ Ho(9.5 nm)/Nb(6.5 nm)/sapphire film. The high-angle X-ray diffraction data confirms epitaxial growth of Nb(110) and Ho(002) on a *a*-plane (110) sapphire. The Inset shows the multilayer structure.

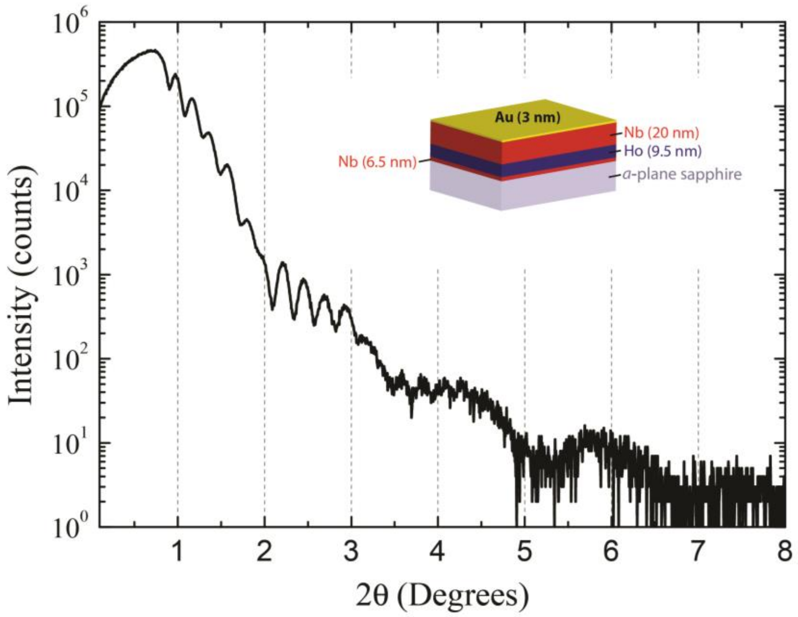

Supplementary Figure 2. Low angle X-ray diffraction data on Au(3 nm)/Nb(20 nm)/Ho(9.5 nm)/Nb(6.5 nm)/sapphire film. Kiessig fringes are observable beyond 6° confirming high quality growth of the layers and a surface roughness of only (approximately) 0.55 nm.

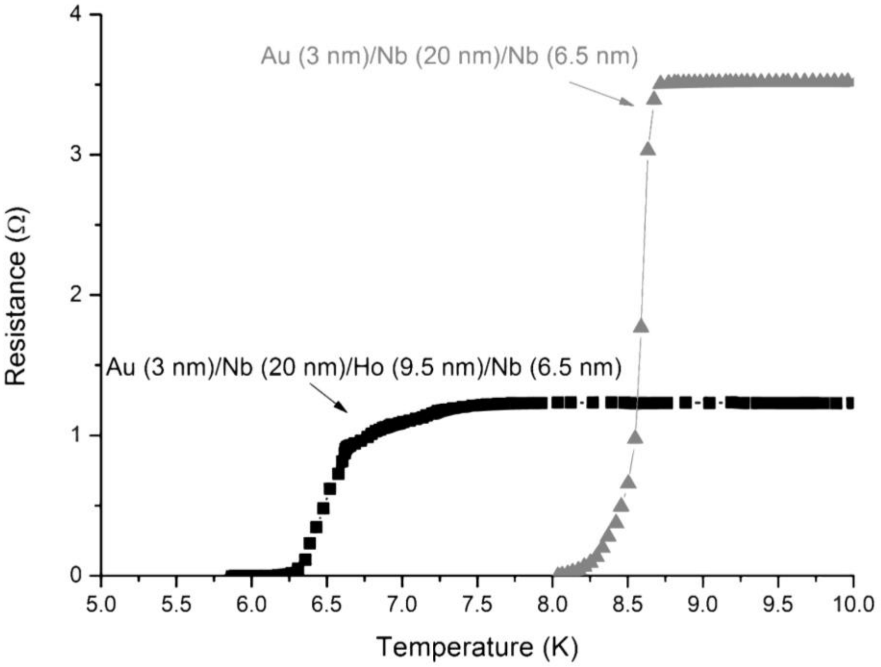

Supplementary Figure 3. Resistance vs temperature (R-T) plots for a Au(3 nm)/Nb(20 nm)/Ho(9.5 nm)/Nb(6.5 nm) film on sapphire and a Au(3 nm)/Nb (20 nm)/Nb (6.5 nm) on sapphire. Electrical transport measurements were performed using a custom-built dipstick probe in a liquid helium dewar in a four-point current-bias setup. From the temperature dependence of a film's resistance, the superconducting transition T_c was defined as the temperature at 50% of the transition. Care was taken to ensure that the applied current (0.1 mA) had no effect on T_c. Critical temperatures of 6.5 K with Ho and 8.5 K without Ho were extracted meaning the proximity effect of Ho reduces T_c by 2 K. The R-T plots were measured in increasing temperature.

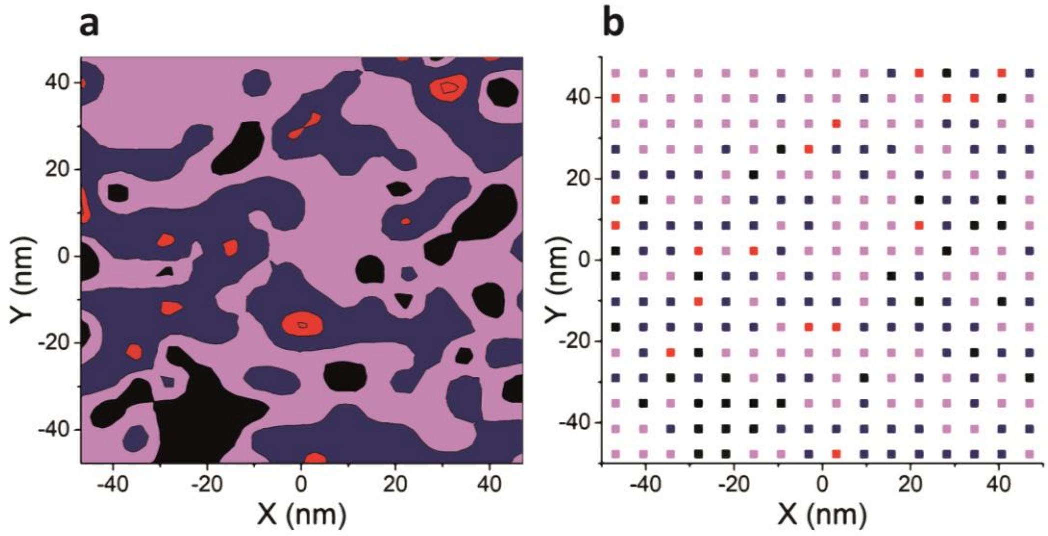

Supplementary Figure 4. Spectral map of Au(3 nm)/Nb(20 nm)/Ho(9.5 nm)/Nb(6.5 nm)/sapphire acquired at 290 mK and corresponding pixelated image. (**a**) Spectral map as reported in the main text (Fig. 3b) and (**b**) corresponding 16x16 matrix of the measured DOS spectra. Each DOS spectrum is classified into one of the four classes shown in Figs. 3c to 3f of the main text and associated with an arbitrary number on a [0, 1] scale (0 for noise, 0.3 for BCS, 0.6 for double-peak and 1 for single-peak). The value 1(0) in this classification represents the extreme of the [0, 1] scale interval in closest (furthest) connection with a signature of odd-frequency superconductivity. The mesh grid in **b** thus obtained is converted into a contour plot through linear interpolation between consecutive points and smoothing of the contour curves to give the spectral map shown in **a**.

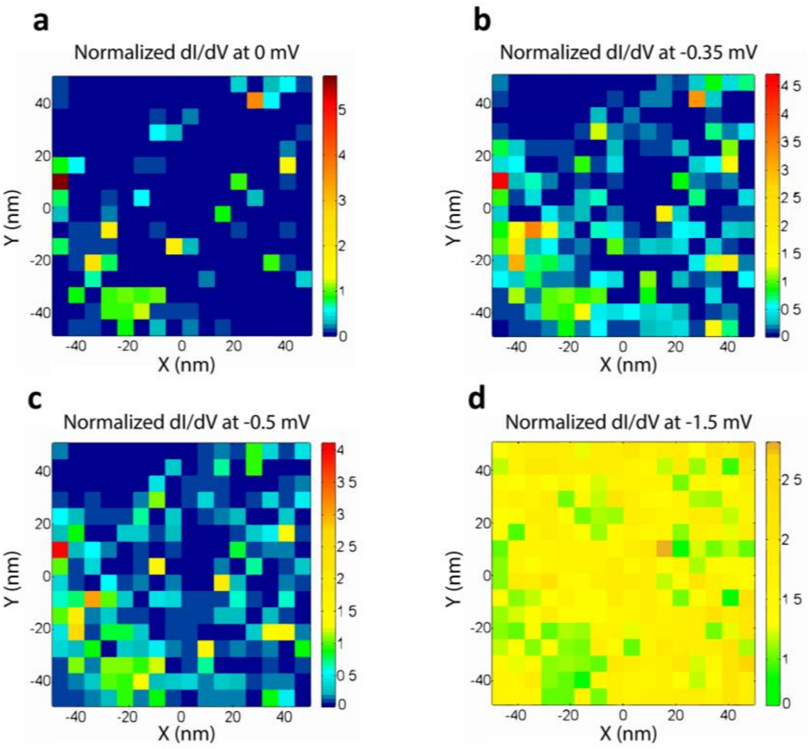

Supplementary Figure 5. **Conductance maps determined at different voltages from the DOS spectra acquired on Au(3 nm)/Nb(20 nm)/Ho(9.5 nm)/Nb(6.5 nm)/sapphire.** Differential conductance (*dI/dV*) maps acquired on the sample area shown in Fig. 3a of the main text at 0 mV (**a**), -0.35 mV (**b**), -0.5 mV (**c**) and -1.5 mV (**d**).

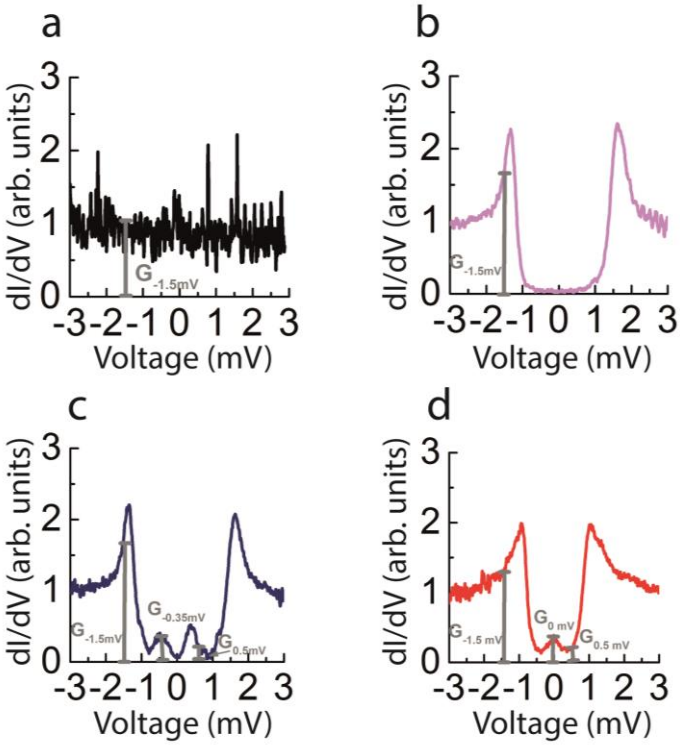

Supplementary Figure 6. **Position of the conductance values at specific voltages in the DOS spectra acquired on Au(3 nm)/Nb(20 nm)/Ho(9.5 nm)/Nb(6.5 nm)/sapphire.** Representative DOS spectra (corresponding to Figs. 3c to 3e in the main text) with marked positions of the conductance values used in the representation of the conductance maps (see Supplementary Note 1).

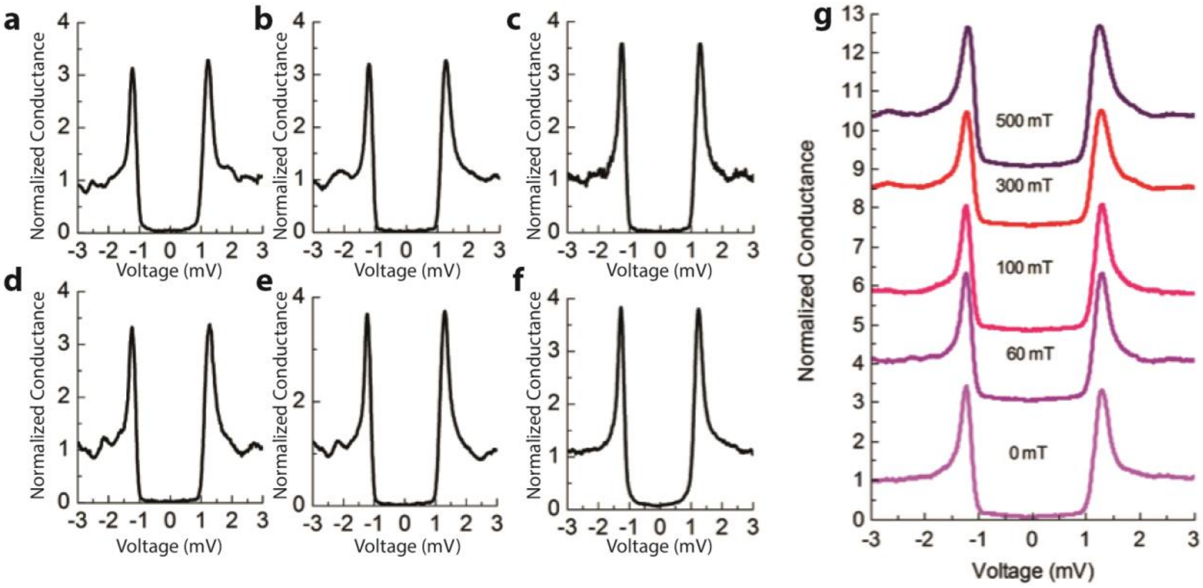

Supplementary Figure 7. Differential conductance spectra for a Au/Nb reference sample and their evolution in an in-plane magnetic field. Control samples were prepared in the same way as the Au/Ho/Nb films as described in the Methods Section of the main text: the Nb is epitaxial and the Au is polycrystalline. No subgap structure was observed on the films. (**a**-**f**) Typical spectra acquired on the Au(3 nm)/Nb(20 nm) control sample at 290 mK. (**g**) Spectra acquired on the same sample with an applied in-plane magnetic field (as labeled); curves are vertically offset for clarity.

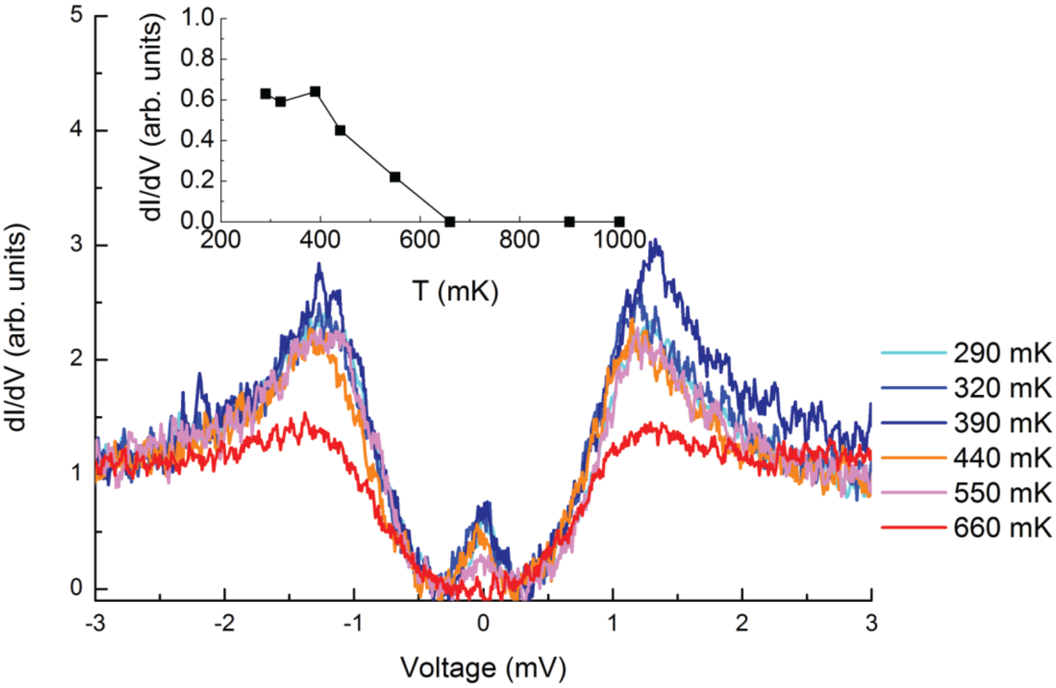

Supplementary Figure 8. Typical temperature dependence of a zero peak acquired on Au(3 nm)/Nb(20 nm)/Ho(9.5 nm)/Nb(6.5 nm) film on sapphire. Evolution of the density of state spectrum with zero peak as a function of increasing temperature and (inset) zero-peak amplitude versus temperature.

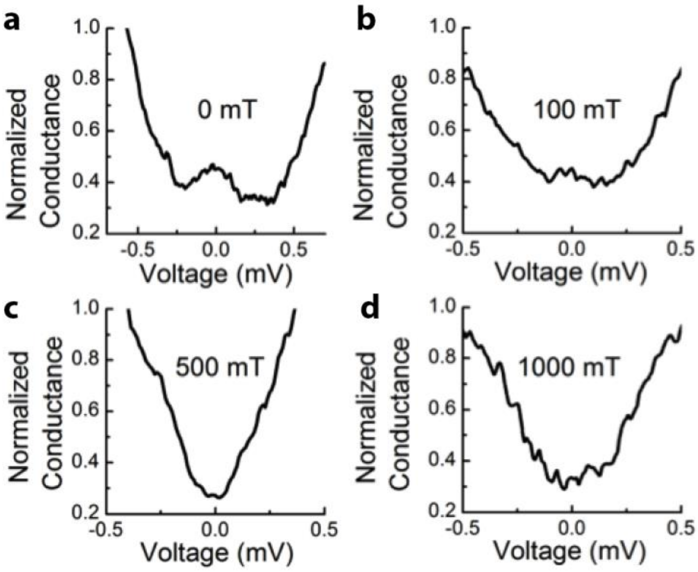

Supplementary Figure 9. Effect of an out-of-plane magnetic field on a zero peak acquired on Au(3 nm)/Nb(20 nm)/Ho(9.5 nm)/Nb(6.5 nm) film on sapphire. The zero peak disappears below 500 mT and does not split into two peaks as expected if the zero peak is related to Kondo effects.

Supplementary Note 1.

To ensure local reproducibility of the spectral features observed in our experiments and to determine any variation of conductivity on sample areas with topographic defects used as a reference, we have determined the conductance maps corresponding to the 16x16 DOS spectra matrix acquired on the 96 nm x 96 nm area of Au/Nb/Ho/Nb sample as given in Fig. 3 of the main text.

The conductance maps have been measured at voltages of 0 mV, -0.35 mV, -0.5 mV and -1.5mV. These specific voltages are necessary to identify the type of spectra (among the four possible ones shown in Figs. 3c to 3f of the main text) corresponding to each of the 16x16 matrix data points for the following reasons:

- The conductance (G) at 0 mV (denoted G_0) can be used to differentiate between a BCS or double-peak spectra where $G_0 \sim 0$, a single-peak or noisy spectra where G_0>>0);

- The conductance value at -0.35 mV (denoted $G_{-0.35}$) corresponds to the maximum conductance of a double-peak subgap feature and it can therefore be used to identify double-peak spectra;

- The conductance value at -0.5 mV (denoted $G_{-0.5}$) for all the DOS spectra that can be related to a double-peak subgap feature ($G_{-0.35}$>>0) can help distinguish between those representative of a rounded U-shaped-like BCS gap ($G_{-0.5} > G_{-0.35}$) and those related to a double-peak ($G_{-0.5} < G_{-0.35}$);

- The conductance at -1.5mV ($G_{-1.5\ mV}$) is normally much larger than 1 for good spectra (double-peak, single-peak, BCS), but close to 1 for noisy spectra.

Supplementary Figure 6 helps visualize the above by showing the positions of G_{0mV}, $G_{-0.35mV}$, $G_{-0.5mV}$, $G_{-1.5mV}$ in the four types of DOS spectra measured in our experiment.

The normalized conductance maps measured at 0 mV, -0.35 mV, -0.5 mV and -1.5 mV are reported in Supplementary Figure 5. The colour scale is the same for all maps, e.g. light green corresponds to dI/dV = 1 in all maps. Unlike for the spectral map reported in Fig. 3b of the main text, which gives a concise overview on the spatial distribution of the different kinds of spectra along with its correlation to the local sample topography, a comparison between corresponding pixels on different conductance maps is here needed to collect the same kind of information.

A few distinct areas, however, are easy to identify in these. For instance, the area around X = -20 nm and Y = -20 nm is clearly noisy, since the conductance is equal to one independently on the voltage, as expected for a noisy spectrum (see Supplementary Figure 6a). The area around X= 20 nm and Y= 10 nm instead is dominated by BCS-like spectra, since the conductance remains very low from 0 mV to -0.5 mV (Supplementary Figures 5a to 5c) and then it becomes significantly larger than 1 at -1.5 mV. Similarly, the maps show that the area around X = 10 nm and Y =-40 nm is characterized by a conductance close to zero at 0 mV, increasing around -0.35 mV and then decreasing again at -0.5 mV – which is clearly indicative of a region where spectra with double-side peaks have been recorded. All these observations are clearly in accordance with the spectral map reported in Fig. 3b of the manuscript and hence confirm its accurateness in describing the spectral distribution on the investigated sample area.

Supplementary Note 2.

To model the experimental data we have performed calculations using the quasiclassical theory of superconductivity in the regime of diffusive transport by using the Usadel equation[1]. The equilibrium properties of the Nb/Ho system under consideration are expected to be well described by the Green's functions g_S and g_F in the superconductor (S) and ferromagnetic (F) regions, respectively. In order to uncover the odd-frequency correlations in Nb, it is necessary to solve the Usadel equation in both the S and F regions in addition to the self-consistent gap equation describing the superconducting order parameter Δ in S. This procedure is more complex than the usual approach in the literature where the bulk solution for g_S is used, allowing for a simplification of the problem as only the Usadel equation in the F region has to be solved.

The two equations to solve for the Green's functions read:

$$D_F\, \partial_x\, (g_F\, \partial_x\, g_F) + \mathrm{i}[\varepsilon\rho_3 + \mathrm{diag}\{\boldsymbol{h}\cdot\boldsymbol{\sigma}, (\boldsymbol{h}\cdot\boldsymbol{\sigma})^{\mathrm{T}}\}, g_F] = 0 \quad (1)$$

$$D_S\, \partial_x\, (g_S\, \partial_x\, g_S) + \mathrm{i}[\varepsilon\rho_3 + \Psi, g_S] = 0 \quad (2)$$

Here, D_F and D_S are the diffusion coefficients in the F and S regions, ε is the quasiparticle energy, $\boldsymbol{h} = \boldsymbol{h}(x)$ is the vector describing the local magnetic exchange field, $\boldsymbol{\sigma}$ is the Pauli matrix vector, while the superscript (T) denotes matrix transpose. The matrices ρ_3 and Ψ read:

$$\rho_3 = \begin{pmatrix} 1 & 0 & 0 & 0 \\ 0 & 1 & 0 & 0 \\ 0 & 0 & -1 & 0 \\ 0 & 0 & 0 & -1 \end{pmatrix}, \psi = \begin{pmatrix} 0 & 0 & 0 & \Delta \\ 0 & 0 & -\Delta & 0 \\ 0 & \Delta^* & 0 & 0 \\ -\Delta^* & 0 & 0 & 0 \end{pmatrix} \quad (3)$$

where $\Delta=\Delta(x)$ is the superconducting order parameter. The notation [...] denotes a commutator. These equations are to be solved with the following boundary conditions at the S/F interface:

$$2\tau_S\, g_S \partial_x g_S = [g_S, g_F] - \mathrm{i}\tau_{S,\varphi}\, [\alpha, g_S] \text{ and } 2\tau_F\, g_F \partial_x g_F = [g_S, g_F] + \mathrm{i}\tau_{F,\varphi}\, [\alpha, g_F] \quad (4)$$

These boundary conditions include the presence of a spin-active interface[2] captured by the parameters $\tau_{F(S),\varphi}$ for a weakly polarized ferromagnet where the matrix α depends on

the orientation of the interface moment relative to the bulk F. The parameter $\tau_{F(S)}$ is a measure of the interface transparency defined as $R_B/R_{S(F)}$ with R_B being the resistance of the barrier region and $R_{S(F)}$ the normal-state resistance of the S(F) regions. At the outer edges of the S and F regions, the boundary conditions read $\partial_x g = 0$. Finally, one also has to take into account the self-consistency equation for the order parameter in the S region:

$$\Delta = \frac{NV}{8} Tr\{\frac{(\rho_1 - i\rho_2)}{2}\tau_3 \int g^K d\varepsilon\} \quad (5)$$

where $g^K = (g_S - g^A)\tanh(\beta\varepsilon/2)$ and $g^A = -(\rho_3\, g_S\, \rho_3)^\dagger$ with $\beta = 1/(k_B T)$, T being the temperature and k_B the Boltzmann constant. The coefficients N and V denote the DOS at the Fermi level in the normal-state of the superconductor and the net attractive interaction between the electrons causing Cooper pair formation. The matrices ρ_1, ρ_2, and τ_3 are 4x4 matrices which are given in Ref. 3.

The above system of equations is solved by an iterative schedule as follows. One first solves the Usadel equation for g_F using an initial guess for the Green's function g_S in the boundary condition for g_F. The resulting solution for g_F is then used to solve the Usadel equation for g_S, which in turn is used as input for the self-consistent equation for Δ. The procedure then starts over using the latest solution for g_S and is repeated until the change in value obtained for $\Delta(x)$ does not exceed a small threshold value ϵ. In this way, one may verify that the obtained solutions for g_F and g_S simultaneously solve the two Usadel equations and the self-consistency equation for the gap.

In our numerical implementation, we made use of the Ricatti-parametrization[4,5] for the Green's function matrix and added a small imaginary value $i\delta$ with $\delta \ll 1$ to the quasiparticle energies ε in order to account for inelastic scattering and pair breaking processes in the bulk S.

Supplementary References

1. Usadel, K. Generalized diffusion equation for superconducting alloys. *Phys. Rev. Lett*. **25**, 507-509 (1970).
2. Cottet, A., Huertas-Hernando, D., Belzig, W. & Nazarov Y. Spin-dependent boundary conditions for isotropic superconducting Green's functions. *Phys. Rev. B* **80**, 184511 (2009).
3. Linder, J., Yokoyama, T. & Sudbø, A. Theory of superconducting and magnetic proximity effect in S/F structures with inhomogeneous magnetization textures and spin-active interfaces. *Phys. Rev. B* **79**, 054523 (2009).
4. Schopohl N. & Maki, K. Quasiparticle spectrum around a vortex line in a d-wave superconductor. *Phys. Rev. B* **52**, 490 (1995).
5. Konstandin, A., Kopu, J. & Eschrig, M. Superconducting proximity effect through a magnetic domain wall. *Phys. Rev. B* **72,** 140501(R) (2005).
